# Macrophage Activation Syndrome: different mechanisms leading to a one clinical syndrome

**DOI:** 10.1186/s12969-016-0130-4

**Published:** 2017-01-17

**Authors:** Claudia Bracaglia, Giusi Prencipe, Fabrizio De Benedetti

**Affiliations:** Division of Rheumatology, Ospedale Pediatrico Bambino Gesù IRCCS, Piazza S. Onofrio 4, 00165 Rome, Italy

**Keywords:** Macrophage activation syndrome, Haemophagocytic lymphohistiocytosis, IL-6, IL-18, IFNγ, Inflammasome, Natural killer

## Abstract

**Background:**

Macrophage activation syndrome (MAS) is a severe complication of rheumatic disease in childhood, particularly in systemic Juvenile Idiopathic Arthritis (sJIA). It is characterize by an uncontrolled activation and proliferation of T lymphocytes and macrophages.

**Main content:**

MAS is currently classified among the secondary or acquired forms of haemophagocytic lymphohistiocytosis (sHLH). The reason is that MAS shares clinical and laboratory features with primary genetic HLH (pHLH). In this context is conceivable that some of the pathogenic mechanisms of pHLH may be involved in other forms of HLH. Heterozygosity for mutations of genes involved in pHLH may lead to a cytotoxic defect and to a development of clinical overt disease. But other different contributors might be involved to the development of MAS such as infections or underlying inflammation. In MAS, the inflammatory status of the patient is a major contributor of the disease. Indeed, the majority of the MAS episodes occurs during active disease phases or at disease onset. In addition, recent evidence in animals and humans suggest that genetics may also play a major role in contributing to hyperinflammation and particularly to macrophages hyper-responses.

**Conclusions:**

We hypothesize that HLH may be one unique clinical syndrome, to whose generation different mechanisms may contribute, and maintained by one final effector mechanism.

## Background

The term Macrophage Activation Syndrome (MAS) identifies a potentially fatal complication of rheumatic diseases. It occurs usually in the context of systemic Juvenile Idiopathic Arthritis (sJIA), but it may occur also, albeit more rarely, in systemic Lupus Erythematosus and Kawasaki disease.

MAS is typically characterized by high fever, lymphoadenopathy, hepatosplenomegaly variably associated with hemorrhages, signs of liver, central nervous system and kidney involvement, and may lead to multiple organ failure. Laboratory abnormalities include decrease in white blood cells, platelet and hemoglobin, hypertransaminasemia, marked increase in ferritin, and evidence for intravascular coagulation activation.

### MAS clinical and laboratory features

Minoia et al. collected in a multinational multicentre study 362 cases of patients with diagnosis of MAS, in the context of sJIA, established by the treating physicians. All patients had fever, a relevant percentage of them (58%) had splenomegaly and lymphadenopathy (51%). Surprisingly, 35% of them had symptoms suggestive of CNS involvement, 16% had pericarditis, which is consistent with systemic JIA, and 20% had clinical evidence of consumption coagulopathy. See Appendix 1: Case 1 for laboratory abnormalities in sJIA and MAS. In the large multinational study, the great majority of the patients had high ferritin. All the patients had liver involvement and, in the great majority of them, transaminases (94%) and LDH (94%) were abnormal. Anaemia (92%) was very frequent; however, thrombocytopenia (70.3%) and neutropenia (54.5%) were not as frequent as in primary HLH (pHLH). Triglycerides (93%) were elevated in the majority of patients; evidence of intravascular coagulation, as demonstrated by elevated d-dimer, was present in the great majority of the patients (97%). Fibrinogen was not below the normal limit in a significant portion of patients (48.6%), as expected in highly inflammatory disease where fibrinogen behaves as a positive acute phase protein [1].

### Classification

In general, MAS is classified among the secondary forms of haemophagocytic lymphohistiocytosis (sHLH), as a sHLH occurring in the context of a rheumatic disease (Table 1), the reason being that HLH and MAS share a number of clinical and laboratory features. The most significant differences are due to the inflammatory background of MAS: particularly platelet counts, as well as fibrinogen levels, are usually higher in MAS compared to pHLH. Primary HLH is caused by mutation in genes that code for proteins that are involved in “perforin-mediated” cytolytic function. They are either proteins such as perforin, directly involved in cytotoxicity, or proteins involved in vesicle transport and fusion with the plasma membrane.

**Table 1:** Classification of primary (genetic) and secondary (reactive) HLH [46–48]

	GENE	FUNCTION
Genetic HLH		
Primary or Familial HLH (pHL)		
pHL-1	Unknown	
pHL-2	PRF1	Pore-forming protein
pHL-3	UNC13D	Vesicle priming (impair granule exocytosis)
pHL-4	STX11	Vesicle transport and fusion
pHL-5	STXBP2	Vesicle transport and fusion
Sporadic associated with Immunodeficiencies		
CHS	LYST	Vesicle transport
GS-2	RAB27A	Vesicle docking
XLP-1/XLP-2	SH2DIA/BIRC4	Signal transduction and activation of lymphocytes
Secondary HLH or Acquired HLH or Reactive HLH		
Infections (EBV, Leishmania, H1N1…)		
Rheumatic diseases (sJIA…)→MAS or rheuma-HLH		
Malignancies (lymphoma…)		

There are several conditions associated with secondary or acquired or reactive HLH. Although definitive epidemiologic data are lacking, sHLH appear to be much more frequent than pHLH. Among infection associated HLH, Epstein Barr Virus is certainly the most common trigger [2]. Additional typical triggers include other Herpes viruses, Leishmania and H1N1 influenz [3–5]. There are a number of cases associated with lymphoma and leukaemia, most frequently T cell receptor gamma-delta bearing T cell lymphomas. These appear to be rare in children, but this occurrence is rather frequent in adults [6]. HLH can also be associated to an underlying rheumatic disease with sJIA being by far the most frequent one. This form of sHLH is what paediatric rheumatologist call MAS or rheuma-HLH. There is a significant number of cases in which an apparent underlying disease or an apparent underlying infection cannot be found [7].

### Genetics of MAS: contribution to defective cytotoxicity

As mentioned above, in pHLH, all causative genes code for proteins involved in the cytotoxic pathway, in which cytolytic granules are transported to and fused with the cell membrane, and eventually pore-forming proteins, such as perforin, are secreted in these immunological synapses [8, 9]. If HLH is considered one clinical syndrome is conceivable that some of the pathogenic mechanisms of pHLH may be involved in other forms of HLH. See Appendix 2: Case 2 for potential impact of heterozygosity for mutations of pHLH genes on development of clinical overt disease and of a defective cytotoxicity [10].

One may also expect that a significant role for genetics may impact on the recurrence of the disease. Indeed, recurrent MAS in patients with sJIA is a well-known phenomenon. However, information on the frequency of recurrences is lacking from the large series reported in recent papers [1, 11–14].

Kaufman et al. performed whole exome sequencing in 14 MAS cases on sJIA and found that 5 out of 14 patients (36%) were heterozygous for at least one mutation of the known genes related to pHLH. In addition, they found that 22 rare variants occurred in at least two patients. Most of these variants code for proteins involved in microtubule organization and vesicle transport [11]. This suggested that cellular assembly and cytoscheletal organization may be involved in the potential defect in cellular cytotoxicity of these patients. A number of other studies have previously looked at the frequency of variants in the genes causing pHLH and overall they all found a number of patients carrying either hypomorphic variants or pathogenic mutations in heterozygosity in both *UNC13d* [15] and *PRF1* [13]. In the context of the Italian National Registry of familial HLH we performed mutation analysis of genes involved in pHLH in 31 patients with sJIA and MAS. The analysis included *PRF1*, *UNC13d*, *STX11*, *STXBP2*and *RAB27a*, and identified monoallelic mutations in 11 of the 31 patients (35.5%). Three out of 31 patients (9.7%) carried a mutation in 2 genes. Interestingly, it has been recently reported that mice carrying heterozygous mutation in more than one gene involved in pHLH carry a significant higher risk to develop HLH following viral infection [16]. Overall, clinical and laboratory feature of MAS in patients carrying the mutations were not different from those of patients who do not carry the mutation. However, recurrences of MAS seem to be more frequent in patients who carry the mutations (mutated 27% versus non-mutated 10%). In our series of patients one patient died because of MAS and she was heterozygous for a known pathogenic *PRF1* mutation (N252S) [17]. Altogether these observations, far from being conclusive, point to the presence of a genetic contribution to MAS similar to that of pHLH. All together these observations suggest that events related to the pathogenesis of pHLH are relevant in sHLH in general, and in MAS in particular: a) accumulation of partial genetic defect in one, or more than one, of the known pHLH related gene b) the possible dominant negative effects of some heterozygous variants [10, 18]. Defective cytotoxicity may impair clearance of infected cells, and/or prolong lymphocyte-APC interactions, both events leading to increased pro-inflammatory cytokine production (e.g. IFNγ) by T lymphocytes [10, 19].

### Different contributors to the development of a unique clinical syndrome

In pHLH the contribution of genetics is so strong that, even in the absence of any demonstrable trigger, genetically caused immune abnormalities are sufficient to drive the individual in full blown clinical HLH. In HLH secondary to infection, in some instances, the strength of the infectious agent is sufficient to cause HLH. Most of the patients who died of H1N1 or H5N1 died with HLH [20]. There are other agents that cause HLH rather often, as mentioned above. In these instances, there must be a contribution from the infectious agent, or more precisely from the response elicited by a given infectious agent. In this respect, it is interesting to note that gene expression profiles of peripheral blood mononuclear cells from patients with symptomatic mononucleosis show evident similarities with gene expression profiles of patients with HLH or MAS [21]. However, there must be also a contribution from the genetic background. Schulert et al. found rare variants in pHLH related genes in 5 out 14 cases of fatal H1N1 influenza infection, all of whom had evidence of hemophagocytosis. Five patients were heterozygous for *LYST* mutation, two of them carried also a *PRF1* mutation [22].

In MAS, the HLH clinical syndrome develops on the background of a highly inflammatory disease such as sJIA. Also in sJIA an infectious trigger appears to play a role. Several MAS appears to be triggered by acute infections in patients with active sJIA. In a recent large multinational survey, this occurrence was documented in approximately 1/3 of the cases [1]. In MAS, it is obvious that an additional contribution must be provided by the inflammatory status of the patient; indeed, the majority of the MAS episodes occurs during active disease phases or at disease onset.

### Contribution of active inflammation to the development of MAS

We generated a model of MAS using the interleukin-6 (IL-6) transgenic mouse (IL-6TG). In these mice, administration of Toll-like receptor (TLR) ligands, including lipopolysaccharides (LPS) and poly(I:C), induces the development of MAS by mimicking an acute infection on a background of high levels of IL-6 [23]. This experimental approach recapitulates what occurs in patients with systemic JIA: an infection triggers MAS on a background of active disease, which is indeed characterized by high levels of IL-6.

Compared to wild type mice, LPS-challenged IL-6 transgenic mice display an increased fatality rate and show hematologic and biochemical features typically present in MAS patients, including lower neutrophils and platelet count, higher levels of ferritin and LDH, as well as a cytokine storm.

When we looked at the stimulatory effect of IL-6, we showed that, in vitro, prolonged exposure of human macrophages to IL-6 leads to increased production of cytokines and chemokines, including Tumor Necrosis Factor-α (TNF-α) and (C-X-C Motif) Ligand 8 (CXCL-8), both at a basal level and upon stimulation with TLR ligands, suggesting a role for IL-6 in amplifying the inflammatory response [24]. In addition, we demonstrated that IL-6 amplifies TLR-induced inflammatory response also in cells originating from inflammatory site, such as synovial fluid mononuclear cells and fibroblast-like synoviocytes isolated from arthritides patients. Indeed, treatments of these cells with IL-6 increased basal production of CXCL8, C-C motif chemokine ligand 2 (CCL2) and Interleukin-1β (IL-1β) following poly(I:C) and muramyl dipeptide stimulation [24].

The other major feature of MAS in the context of sJIA is the transitory defect in cellular cytotoxic function. Grom et al. demonstrated that most of the sJIA patients have profoundly decreased Natural Killer (NK) cell activity [25]. The reduced NK cytotoxic activity observed was associated, in some patients, to a decreased perforin expression on NK cells and, in other patients, to a decreased perforin expression on T-cells. However, overall, the great majority of patients display a NK cytotoxic activity defect.

Consistently, we found that NK activity was impaired also in the murine model of MAS, the IL-6TG. Indeed, IL-6TG mice challenged with poly(I:C), a substitute for viral dsRNA, display a significant decrease in NK cell cytotoxicity, associated to lower expression levels of perforin and granzyme B, in the absence of altered granule exocytosis [26]. An example of IL-6 leading to decreased cytolytic activity of lymphocytes on top of an underlying genetic defect has been shown [10]. Similarly, in vitro experiments revealed that exposure of human PBMCs to high levels of IL-6, such as those typically present in sJIA, inhibits NK cell cytotoxicity by down-regulating the expression of perforin and granzyme B. Altogether, this evidence suggests that high circulating levels of IL-6 in JIA patients, besides their role in increasing macrophage activation and TLR response, play a major role in inducing a transitory defect in cytotoxicity on top of a subclinical genetic defect.

In addition to IL-6, other inflammatory cytokines may play a role in predisposition to MAS. Indeed, even in patients treated with tocilizumab, in which IL-6 was fully neutralized, MAS may occur [27]. One of the potential candidates is IL-1β. There is a number of observations pointing to the efficacy of high dose of anakinra in at least some patients with MAS [28, 29]. On the other hand, there is a number of studies describing patients who developed clinically overt MAS while being treated with both IL-1 blockers anakinra and canakinumab [30–32]. In apparent contrast, in criopyrinopathies, which are the purely IL-1β mediated, cases of MAS are not described. Overall, these data would suggest that neither IL-6 nor IL-1β alone may be the major contributors to the predisposition to MAS.

Increasing number of evidence suggests that interleukin-18 (IL-18) might be another relevant mediator in sJIA. In a study performed in Japan, the subset of sJIA patients characterized by high levels of IL-18 (and conversely low levels of IL-6) had a higher frequency of MAS episodes [12]. These data need to be confirmed in a Western population. Albeit in a small number of patients, data from Put et al. are consistent with this observation: in sJIA patients high IL-18 levels do not markedly increase during active MAS, pointing to IL-18 being a predisposing cytokine [33]. The mechanisms behind the predisposing effect of IL-18 are not clear yet. It is well know that IL-18 affects NK cell activity, but preliminary evidence suggests that it may also regulate macrophage responses [34].

### Contribution of genetics to hyperinflammation

In addition to the known role of genetics of cytotoxicity genes in HLH, recent evidence in animals and humans suggest that genetics may also play a major role in contributing to hyperinflammation and particularly to macrophages hyper-responses. Indeed, data in the animals clearly support this hypothesis. The most studied model of genetically driven HLH are perforin knock-out mice which, upon LCMV infection, develop full blown HLH and die [35]. When *UNC13d* deficient mice are crossed with mice deficient for MYD88, which is a central element in TLR intracellular signalling, the animals are protected from disease development, demonstrating that, at least in animals, TLR signalling and macrophage responses are crucial [36]. Indirect evidence in humans has been provided by a study suggesting that a polymorphism in IRF5, a transducer of intracellular TLR, is associated with the risk of MAS in patients with sJIA [37]. In addition and much more importantly, direct evidence in humans is provided by the recent observation that gain of function mutations of NLRC4, an inflammasome sensor, cause a disease characterized by recurrent or dramatic HLH. This observation demonstrates in humans that genetically induced abnormalities in autoinflammation cause HLH. The disease, still described in very few patients appear to be characterized by early onset of fever, skin rash, severe gastro-intestinal involvement, splenomegaly and recurrent, severe MAS/HLH [38, 39]. Immunological studies revealed that the most striking abnormality is represented by surprisingly high levels of IL-18 pointing to the fact that the NLRC4 protein is particularly important in regulating IL-18 production and further supporting the role of IL-18 as a predisposing factor to HLH/MAS [38].

### Different contributors and one pathogenic mediator?

It is reasonable to hypothesize that several factors, acting at different steps, contribute to the development of MAS and sHLH. A multilayer model of pathogenic events leading to the development of MAS has been proposed, including genetics, inflammatory activity and infection, contributing to reaching a threshold of activation that results in overt MAS [40]. In perforin knock-out mice infected with LCMV, the most used mouse model of primary HLH, neutralization of a number of cytokines does not produce any relevant clinical effect. However, neutralization of IFNγ leads to protection from disease and to survival of the animals [35]. Based on a number of experiments both in mice and on human cells, it has been hypothesized that mutations of genes impacting on cytotoxic activity generate an inability of cytotoxic T cells to lyse the target cells. This defect leads to prolonged hyperactivation of T cells with hyperproduction of IFNγ [10, 19]. High levels of IFNγ leads to activation of macrophages, as a compensatory mechanism aimed at phagocytosis of the infected cells. Indeed, these patients do not succumb from overwhelming infections. These activated macrophages produce very large amounts of inflammatory cytokines and generate a cytokine storm, which is the driver of the clinical and laboratory features of HLH and eventually leads the host to death, if left untreated.

Translating from pHLH to MAS and based on the similarities in clinical and laboratory features as well as in genetic contribution, we have recently investigated whether IFNγ could potentially be involved also in MAS. IFNγ levels were found to be higher in patients with active MAS at sampling compared to patients with active sJIA without MAS at sampling [41]. Using the above mentioned mouse model of MAS in IL-6 transgenic mice we have also found that neutralization of IFNγ improved survival and reverted biochemical abnormalities (Prencipe G. 2016 submitted). Interestingly, in patients with MAS a marked increase in CXCL9 levels was also found. CXCL9 is known to be specifically induced by IFNγ [42]. Indeed in patients with active MAS levels of IFNγ and CXCL9 were strictly related to laboratory features of MAS, including ferritin levels, white blood cell and platelet counts, and LDH [41]. These correlations were not present in patients with active sJIA without MAS. These data suggest that during MAS, but not during active sJIA, there is a specific activation of IFNγ and of the IFNγ pathway. Noteworthy in the NLRC4 patient, reported by Canna et al., one of the cytokines that increases significantly during active HLH episodes is indeed CXCL9 [38]. We also found high levels of CXCL9 in a patient with NLRC4 induced disease (unpublished). Observations in MAS and in NLRC4-induced disease show that hyperinflammation induced by dysregulated inflammatory responses may directly cause overproduction of IFNγ.

## Conclusion

All together these observations are consistent with the hypothesis that HLH may be one clinical syndrome maintained by one effector mechanism, which involves increased macrophage activation and increased T-cells activation. In this loop, IFNγ seems to play a major role. In classical pHLH, genetic mutations and the consequent defect in cytotoxic pathways are sufficient to drive a patient above the threshold eliciting this vicious loop. NLRC4-induced disease provides proof of concept that genetic predisposition to inflammasome hyper-response alone can drive patients directly into the same vicious loop. In patients with MAS an additive/synergist role is played by several contributors, including infectious triggers, genetics, of both cytotoxicity and inflammatory response, and inflammatory activity of the underlying rheumatic disease. Recently generated evidence points to IFNγ as the major mediator also of HLH secondary to infections [43, 44], as well as of MAS. These findings may have significant translational value. A monoclonal antibody to IFNγ is in clinical trial in primary HLH with initial satisfactory response [45] and a trial in MAS is being planned.

## Appendix 1: Case 1

### **Case 1**

An 8 years old Caucasian boy with sJIA onset one year before, suddenly developed persistent fever, generalised seizures, hypotension, purpura and splenomegaly. Laboratory picture was completely different if compared to his laboratory at sJIA onset. Blood count showed leukopenia with neutrophenia and anemia. Erithrocyte sedimentation rate (ESR) was relatively low, however CRP was high as it was. Fibrinogen was low because of intravascular activation of the coagulation, d-dimers were high. He presented also liver involvement with some degree of liver insufficiency. The most striking feature was hyperferritinimia, which was markedly higher compared to sJIA presentation (Table 2). This case represents a typical picture of MAS complicating sJIA. Changes in laboratory features are the hallmark and these have been recently mathematically characterized in multination effort [1]. However, MAS may represents a significant challenge when occurring at sJIA onset.

**Table 2:** Patient’s laboratory features

Laboratory Features	sJIA presentation	MAS presentation
White blood cells, x10^9^/L	15.04	3.02
Neutrophils %	86%	19%
Haemoglobin, g/dL	9.08	7.09
Platelet, x109/L	640	125
Ferritin, ng/mL	880	11345
ESR, mm/h	98	32
CRP, mg/dL	13.08	19.07
Fibrinogen, mg/dL	560	88
D-dimers, ng/mL	<10	>40
Sodium, mEq/L	136	129
ALT, U/L	23	180
AST, U/L	18	145
LDH, U/L	380	1910
Bilirubin, mg/dL	0.06	2.06

ESR, erythrocyte sedimentation rate; CRP, C-reactive protein; ALT, alanine aminotransferase; AST, aspartate aminotransferase; LDH, lactate dehydrogenase

## Appendix 2: Case 2

### **Case 2**

A 15 years-old, Caucasian male presented with persistent fever since 4 weeks, diarrhoea vomiting, skin rash, hepatosplenomegaly, and elbows arthritis. Blood tests showed high ferritin (5896 ng/ml), liver inflammation (AST 224 IU/l, ALT 635 IU/l, LDH 676 IU/l), and intravascular activation of coagulation with high d-dimers (2.26 μg/ml), and a bone marrow biopsy showed hemophagocytosis. He was treated with glucocorticoids, cyclosporine A and cyclophosphamide with good response. Three years later, he presented again with persistent fever, skin rash, diffuse arthralgia, and myalgia. He referred to the malaise and arthralgia as being the “same disease” as the one 3 years before. As soon as ferritin started to rise significantly (5481 ng/ml), dexamethasone and cyclosporine A were started with a prompt response. He was found to carry a single copy heterozygous missense mutation in *RAB27a*. This same mutation was found in his father, who was apparently healthy, but who had a baseline moderately high serum ferritin level (800 ng/ml). Functional tests showed a marginal defect in CD107a degranulation in the patient and his healthy father [10]. Indeed experiments with this very mutation have shown an impact on cytotoxic function as well as on the duration of the immune synapses [10]. This patient appears to have had two episodes of HLH, which cannot be strictly defined as primary or genetic HLH; it is however, tempting to speculate that carriage of this mutation plays an important role in the two recurrences of secondary HLH.

## Abbreviations

CCL2: C-C motif chemokine ligand 2
CXCL8: (C-X-C Motif) Ligand 8
CXCL9: (C-X-C Motif) Ligand 9
HLH: Haemophagocytic lymphohistiocytosis
IL-18: Interleukin-18
IL-1β: Interleukin-1β
IL-6: Interleukin-6
IL-6TG: IL-6 transgenic mouse
IRF5: Interferon regulatory factor 5
LCMV: Lymphocytic choriomeningitis virus
LPS: Lipopolysaccharides
MAS: Macrophage activation syndrome
MYD88: Myeloid differentiation primary response 88
NK: Natural killer
PBMC: Peripheral blood mononuclear cell
pHLH: Primary or familial haemophagocytic; lymphohistiocytosis
sHLH: Secondary or acquired haemophagocytic lymphohistiocytosis
sJIA: Systemic juvenile idiopathic arthritis
TLR: Toll-like receptor
TNF-α: Tumor necrosis factor-α
